# Editorial: Harnessing artificial intelligence for multimodal predictive modeling in orthopedic surgery

**DOI:** 10.3389/fsurg.2025.1702415

**Published:** 2025-10-10

**Authors:** Babak Saravi, Sebastien Couillard-Despres, Gernot Lang, Frank Hassel

**Affiliations:** 1 Department of Oral, Maxillofacial and Facial Plastic Surgery, Medical Faculty and University Hospital Düsseldorf, Heinrich-Heine-University Düsseldorf, Düsseldorf, Germany; 2 Department of Orthopedics and Trauma Surgery, Faculty of Medicine, Medical Center - University of Freiburg, University of Freiburg, Freiburg, Germany; 3 Department of Spine Surgery, Loretto Hospital, Freiburg, Germany; 4 Institute of Experimental Neuroregeneration, Paracelsus Medical University, Salzburg, Austria; 5 Austrian Cluster for Tissue Regeneration, Vienna, Austria

**Keywords:** artificial intelligence, multimodal predictive modeling, orthopedic surgery, machine learning, deep learning, computer-aided surgery, clinical decision support, translational medicine

Orthopedic surgery is becoming a data-dense discipline (1). Clinical records, perioperative physiology, radiological imaging, and patient-reported outcomes now coexist in routine care, yet they are rarely interpreted together at scale. Artificial intelligence (AI)—particularly when applied to multimodal inputs—offers a way to synthesize this information for better diagnosis, risk prediction, and treatment planning (2). This Research Topic brings together eight contributions spanning imaging analytics, perioperative risk modeling, computer-aided and robot-assisted surgery, and a narrative review of large language models (LLMs) in orthopedics. Collectively, they map where the field is today and where it needs to go next.

## From unimodal to multimodal prediction

Traditional approaches in orthopedics have leaned on single data streams—isolated imaging reads, perioperative labs, or clinician scores—yielding only partial views of complex disease. Multimodal models instead couple two or more streams, for example radiological imaging with biomechanical markers, surgical variables, and patient-reported outcomes, within integrated frameworks. When designed with complementary signal in mind, such systems improve robustness and deliver predictions that are more clinically actionable—covering progression risk, implant survivorship, and functional recovery trajectories. This pivot from unimodal to multimodal is not only technical; it requires common data models, harmonized labeling, and evaluation protocols that reflect real clinical decisions.

## From risk prediction at the bedside to deployment-ready tools

Sun et al. present an externally validated machine-learning model to predict perioperative blood transfusion in patients with osteonecrosis of the femoral head undergoing total hip arthroplasty. Using feature selection with LASSO and correlation analysis, nested resampling across four algorithms, and a clinician-friendly logistic-regression nomogram, the authors report strong discrimination on both internal and external datasets—an encouraging step toward pragmatic adoption and better stewardship of blood products Sun et al.

## What “multimodal” should mean in imaging pipelines

Yayli et al. compare single-model and multi-model deep-learning strategies for Kellgren–Lawrence grading on 14,607 knee AP radiographs cropped by a YOLOv5 detector. The finding that a well-tuned single model outperformed a more complex multi-model pipeline—and that CLAHE preprocessing usually harmed performance—reminds us that “more modalities” and “more moving parts” do not automatically translate into better clinical classifiers. Task-specific architecture selection and careful preprocessing remain paramount Yayli et al.

## Imaging labels grounded in clinical reality

Liu et al. build multicenter MRI-based models to identify calcified lumbar disc herniation, using CT as the reference standard. A ResNet-34 classifier achieved high test accuracy and strong external-validation AUC, illustrating how AI can elevate everyday MRI reading while anchoring labels to harder-to-obtain but more definitive imaging. The study exemplifies rigorous model development with clear pathways to generalization Liu et al.

## Surgical innovation as both subject and substrate for AI

Yang et al. compare tibial transverse transport with periosteal distraction for refractory diabetic foot ulcers. Both approaches healed all wounds; periosteal distraction required less operative time and blood loss while achieving comparable limb perfusion improvements. Although not itself an AI study, this work highlights standardized clinical endpoints and physiologic readouts—precisely the outcomes that future multimodal prediction systems should target and help personalize Yang et al.

## Protocolizing translation: prospective, external, and clinician-comparative evaluation

Xi et al. describe a protocol to predict bone cement leakage (and its subtypes) during percutaneous kyphoplasty by directly analyzing preoperative CT and MRI, with internal retrospective development, a prospective internal test, and an external multicenter cohort—plus head-to-head assessment against clinicians. This is the kind of translational design that will be required for trustworthy deployment in spine care Xi et al.

## Digital planning that already moves the needle

Cheng et al. show that computer-aided design (CAD) in preoperative planning for total hip arthroplasty improves implant sizing accuracy and reduces blood loss, operative time, and length of stay—tangible patient-relevant outcomes. As CAD workflows increasingly interoperate with predictive models (e.g., automated templating and component selection), the line between “planning tool” and “AI copilot” will continue to blur Cheng et al.

## Personalized surgical planning and beyond

Multimodal AI opens the door to individualized planning. By combining preoperative imaging with gait analysis, musculoskeletal simulation outputs, and relevant demographics, models can inform implant selection, surgical approach, and rehabilitation pathways. The anticipated benefits—lower revision rates, improved function, and higher patient satisfaction—depend on embedding these tools in the systems surgeons already use (templating/CAD, navigation), with transparent rationale and guardrails that support shared decision-making.

## Robotics as a data engine for learning systems

In a comparative series, Chen et al. report that robot-assisted retrograde drilling for osteochondral lesions of the talus is safe, minimally invasive, and achieves short-term outcomes comparable to arthroscopic microfracture while reducing operative time. Beyond the clinical result, robot navigation creates structured intraoperative data and kinematic logs—rich streams that future predictive models can leverage for procedural guidance and outcome prediction Chen et al.

## LLMs in orthopedics: promise with necessary guardrails

Giorgino et al.'s narrative review synthesizes early experience with ChatGPT in orthopedics—from patient education and triage to clinical decision support—while candidly discussing limitations, bias, privacy, and the need for human oversight. For multimodal prediction specifically, LLMs may serve as user interfaces that explain risk, elicit patient preferences, and translate model outputs into shared decisions—so long as we preserve verification, transparency, and accountability Giorgino et al.

## Cross-cutting lessons from this Research Topic

1. Purposeful multimodality. Adding channels (e.g., clinical variables to imaging) must be hypothesis-driven and demonstrably additive; parsimony can outperform complexity when inputs are redundant, as shown in knee OA grading Yayli et al.
2. External validation and prospective testing are non-negotiable. Several contributions move beyond single-center internal splits, an essential step toward generalizable tools Liu et al.
3. Human-centered deployment beats model-centric reporting. Nomograms, CAD templates, and robot-navigation interfaces are how predictions actually reach clinicians Sun et al.
4. Reliability over peak AUC. Calibration, decision-curve utility, and inter-rater consistency—especially vs. clinician performance—matter for adoption; protocolized evaluations are the way forward Xi et al.
5. Ethics and governance remain foundational. Bias mitigation, privacy-preserving data linkage, and auditability must be embedded from the outset, as emphasized for LLMs Giorgino et al.

## Looking ahead: critical challenges and practical solutions

- Data quality and scarcity. Orthopedic datasets are fragmented and heterogeneous. Priorities include multi-center curation under shared ontologies; linkage of DICOM imaging to structured perioperative variables and PROMs; and judicious use of augmentation, synthetic data, and transfer learning to improve generalizability. Pre-register prospective, multicenter evaluations and report calibration and decision-curve utility—not accuracy alone.
- Integration and interoperability. Robust multimodal modeling requires interoperable platforms: standardized data formats (e.g., DICOM-Seg/RT, FHIR), vendor-neutral archives, and APIs that allow safe model invocation inside PACS, templating/CAD, and robot navigation. Designing for workflow—clear UI, latency budgets, and audit trails—matters as much as model architecture.
- Ethics and regulation. Ensure algorithmic transparency, appropriate consent pathways, and bias/fairness audits across subgroups. Maintain versioning, post-deployment monitoring, and explainability commensurate with the decision at hand. Communicate risk in ways that support shared decisions among clinicians and patients.

Taken together, these advances point toward a learning orthopedic healthcare system in which routinely captured outcomes continuously update and recalibrate multimodal models—closing the loop from data to decision to better care.

We thank all authors, reviewers, and the editorial office for their contributions to this Research Topic and for advancing rigorous, clinically oriented AI in orthopedics. The work assembled here demonstrates concrete progress—from bedside risk tools to protocolized multimodal imaging and digitally enabled surgery—while underscoring the standards needed for safe, equitable, and effective translation into practice.
